# Volatilisation Behaviour and Mechanism of Lead-Containing Slag during Physical Property Tests

**DOI:** 10.3390/ma17071531

**Published:** 2024-03-27

**Authors:** Wen-Dan Tang, Jun-Xue Zhao

**Affiliations:** School of Metallurgical Engineering, Xi’an University of Architecture and Technology, Xi’an 710055, China

**Keywords:** lead slag, volatilisation, volatiles, slag-phase reaction

## Abstract

According to physical property tests of lead-containing slag, the volatilisation behaviour of lead slag will have adverse impacts on test accuracy and further affect the control of chemical reactions, solidification and removal of inclusions during smelting. To analyse the volatilisation characteristics of lead slag, in this paper, four kinds of lead slags from lead direct reduction smelting with different PbO and ZnO content are taken. thermogravimetry, ISP-TOF were used. Additionally, the changes in volatiles and slag composition and phases were analysed with XRD and ICPS, and the volatilisation reaction mechanism was discussed. The results indicated that the volatilisation of lead slag can lead to a big weight loss of about to the slag with higher PbO content. The weight loss increases with the PbO content in slag increases. The volatile corresponding to the weight loss above 900 °C is mainly PbO and less ZnO. The higher the temperature is, the stronger the volatilisation is. With the increase in temperature and keeping time, most of the PbO can be evaporated and leaves little PbO in the residual slag. This will has great effect to physico-chemical property measurement of the slag with higher PbO content, especially to the property measurement that be kept at high temperature for a long time. Because the volatiles is trend to condense with the temperature decrease, mass spectrometer is limited by the condensation of volatiles, i.e., PbO, ZnO and so on, in the connection pipeline. The device should be modified for this use.

## 1. Introduction

Lead-containing slag is the intermediate product of the lead smelting process and contains Pb and Zn. The Pb concentration continues to decrease with smelting progress, so Pb slag is divided into low-lead slag and high-lead slag [1,2,3,4]. Pb and Zn are easily converted into volatile substances such as PbO(g), Zn(g) under reasonable conditions [4,5,6,7]. These substances volatilise at high temperatures and form heavy metal dust [8,9]. In different reaction systems, the volatilisation mechanisms of lead-containing slag are different. For example, the oxygen potential not only affects the existence of volatiles but also affects the activation energy of volatilisation reactions [4], which may lead to nonlinear variations in the rate of volatilisation reactions. Moreover, the contents and diffusion conditions of volatiles also significantly impact the volatilisation rate [10]. In physical property tests, including slag melting temperature and viscosity tests, continuous volatilisation will change the composition and properties of the slag, thereby adversely affecting the test results. There is no unified standard for evaluating the degree of impact [11,12]. PbO-ZnO-Fe_3_O_4_-SiO_2_-CaO slag is a typical product of the lead smelting process [13,14]. Research on the volatilisation mechanism of Pb/Zn dust is beneficial for improving the accuracy of measurements of slag composition and physical properties during lead smelting, as is studying the generation mechanism of this material.

M. Perez-Labra et al. [15] studied the change process of the liquid phase in a high-lead and low-lead slag system, successfully predicted the temperature range of the generation of calcium and lead silicates, and confirmed the required temperature and composition conditions for the generation of Ca_2_ZnSi_2_O_7_ and PbFe_12_O_19;_ however, the impact of the volatilisation of Pb and Zn on the physical phase reaction was unknown. Yanling Zhang et al. [6] examined the volatilisation characteristics of low-lead slag at high temperature and measured the volatilisation characteristics of different lead-containing compounds. Research results show that the volatilisation capacity of PbO is lower than that of PbCl_2_. However, as an intermediate product of volatilisation reactions, the proportion of PbO has a great effect on the total volatilisation. Yaru Cui et al. [10] investigated the volatilisation characteristics of high-lead slag during physical property tests. Their study revealed that when the temperature exceeded 900 °C, the volatilisation proportion of PbO(s) exceeded 50%, and diffusion conditions were the main influencing factor. From the above research analysis, it is concluded that the role of PbO(s) in slag is complex and is influenced by various factors, such as the slag composition, diffusion conditions, and reaction atmosphere. The specific volatilisation mechanism of Pb in slag systems is not fully clear.

Theoretically, ZnO(s) do not have volatile properties, and Zn(s) should not exist in a nonreducing atmosphere. However, after the slag is heated, there is still a loss of Zn. Under similar atmospheric conditions, such as during the oxidation stage of the direct lead smelting process, molten lead slag also produces Zn-containing dust. Herein, it is speculated that Zn(s) must be involved in volatilisation, but the specific transformation process of Zn remains to be explored. Research by Si-Jia Wang [16], Zhang [17,18], Hiroyuki Nakada [19] and Mijatović [20] revealed that the production of Zn dust is also restricted by CaO and SiO_2_, which are similar to Pb-containing dust. The volatilisation rate of Zn(s) decreases over time. This suggests from another perspective that ZnO(s,l) will react with other components and that Zn(s,l,g) may come from slag-phase reaction. Similar reaction models can be found in blast furnaces, converters, and electric furnaces [21,22,23,24]. Among them, the Fe-ZnO-FeO system can be considered, and Fe or FeO are the reducing agents. The studies mentioned previously have successfully identified the source of Zn(s); however, the detailed mechanisms underlying Zn transformation and volatilisation remain unclear.

This paper examines the PbO-ZnO-Fe_3_O_4_-SiO_2_-CaO system, which varies from 3% to 40% in PbO content and from 13% to 6% in ZnO content, as the subject of research. The volatilisation patterns of slag and the phase transformation of valuable metals were investigated. This is achieved by measuring the slag volatilisation rate and the volatiles produced, as well as by analysing the related reaction processes. The findings offer theoretical support for the examination of slag compositions, the accuracy of determining physical property tests in metallurgical processes, and the mechanisms behind the formation of Pb/Zn-containing dust.

## 2. Experimental Setup and Procedure

### 2.1. Experimental Materials

The raw materials used in the experiment, PbO, SiO_2_, CaO, CaCO_3_, ZnO, and Fe_3_O_4_, were all analytically pure powders (Sinopharm Chemical Reagent Co., Ltd., Shanghai, China). The purity of Fe_3_O_4_ was more than 85.75%, the purity of CaO was more than 98%, as verified by XRD and XRF, and the other reagents were more than 99% pure.

The slag sample composition is chosen based on the practical Pb smelting—reduction step. In this step, the PbO content in slags is gradually reduced from about 40% to 2.69% [25,26]. With the reduction of PbO, the other components content increase in accordance. The ZnO content changes from 13% to 6%.

In this test, all the slag samples are prepared with the relevant agents and the composition is shown in Table 1.

After the samples were prepared proportionally, the samples were mixed and ground for 1 h in an agate mortar and sealed in a sample bag for later use. Before each use, the samples were dried in a 105 °C air drying oven for 4 h.

### 2.2. Experimental Apparatus and Procedure

A simultaneous thermal analyser (TG-DTA1750, Setaram Evo, France) was used for thermal analysis. Samples #1–4 and #6 chemically pure substances corresponding to samples weighing 10–11 mg were placed into an alumina crucible for STA. The maximum temperature was 1380 °C, and the heating rate was 10 °C/min.

The detection of escaped substances was conducted using an in-situ pyrolytic time-of-flight mass spectrometer (ISP-TOF, Xiamen University, Xiamen, China), which can in-situ detect substances with different mass–charge ratios through TOF at high temperatures. The details are shown in [27]. The lead sample was placed in an alumina tube and then placed in a heating chamber. The lead sample was heated to 1100 °C (max temperature) by an electric heating wire. The testing chamber was connected to the heating chamber by a 3 mm long stainless steel pipe. The test results are recorded every 50 milliseconds.

**Figure 1:**
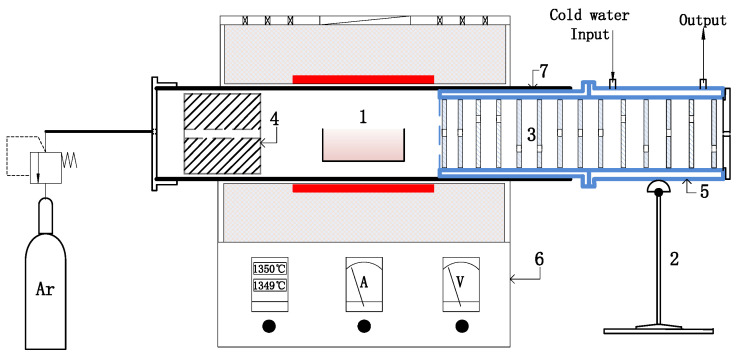
Schematic diagram of the collection devices. 1—Sample; 2—Support bar; 3—Steel plate with hole; 4—Steel plate with hole; 5—Condenser shell; 6—Firebrick with hole; 7—Furnace tube.

To get the volatilisation characters at higher temperature, a tubular furnace was used to evaporate the lead slag, and the near maximum volatilization rate of the lead slag at 1380 °C was determined. The specific structure of the test system is shown in Figure 1.

The argon flow rates used in the experiment were 1 L/min (<950 °C) and 1.5 L/min (≥950 °C). The heating rate was 10 °C/min. The holding time was 20 min and the temperature was held at 1350 °C. The internal dimensions of the crucible were 10 × 2.5 × 2.5 cm, and the thickness of the crucible was 3–3.5 mm. The volume was 10 mL. The volatiles were collected in the condenser and then taken for examination after the experiment.

The experiment was conducted in two groups. In the first group experiment, volatiles were obtained. The sample was placed in the tube furnace, and argon gas was applied. The furnace temperature was increased to 1350 °C at a heating rate of 10 °C/min, after which the mixture was kept at this temperature for 8 h. When the furnace temperature dropped below 900 °C, the condenser was removed, the collected condensed material was obtained, and the volatiles were stored in a sealed container. In the second experiment, every slag sample was heated to a designed temperature and then maintained at the temperature for 15 min. Finally, the furnace temperature was reduced to the normal temperature within >12 h to obtain the cooled slag sample for further examination. The designed temperature is at 850 °C, 950 °C, 1050 °C, 1150 °C, 1250 °C, and 1350 °C respectively.

The volatile matter collected was detected by X-ray diffraction (D8 Advance, Bruke, Bremen, Germany) and inductively coupled plasma–mass spectrometry (5110, Agilent, Santa Clara, CA, USA). X-ray diffraction was used to analyse the phase of the residual slag.

## 3. Results and Discussion

### 3.1. Weight Loss of Lead Slag at High Temperatures

**Figure 2:**
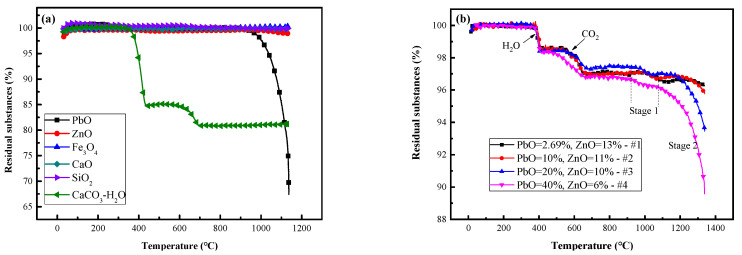
Weight losses of 6 pure substances at 30–1200 °C (**a**) and #1–4 lead slags at 30–1380 °C (**b**).

Figure 2 shows the weight loss curves of the pure material and slags #1–4.

The weight loss of each pure component in an inert atmosphere can be seen in Figure 2a. ZnO, Fe_3_O_4_, CaO and SiO_2_ almost never lose weight. CaCO_3_·xH_2_O lost weight twice at 300–410 °C and 600–720 °C by losing 25% of the total mass of crystalline water and 4.1% of the total mass of CO_2_, respectively. The PbO content tended to decrease by 37.5% from 880 °C to 1150 °C.

The weight loss curves of samples #1–4 are shown in Figure 2b. For samples #1–4, The total mass loss was 3.68%, 4.18%, 6.54%, 10.39%. The crystalline water loss was approximately 1.43%, 1.42%, 1.57%, and 1.7%, respectively, and the CO_2_ loss was approximately 1.62%, 1.61%, 1.18%, and 1.58%, respectively. H_2_O and CO_2_ are derived from CaCO_3_·xH_2_O. This is because CaO deteriorates into CaCO_3_·xH_2_O partly during the mixing and grinding process. The varying trend of the crystalline water content in samples #1–4 is opposite to that of the CaO content in the raw slag. The variation trend of CO_2_ is the same as that of CaO, but the data for #3 are abnormal. This difference indicates that there is some reaction causing the abnormal consumption of H_2_O. After 900 °C, there are two weight loss zones for the four samples in the high-temperature process, namely, the Stage 1 zone from 920–1080 °C and the Stage 2 zone from 1080–1380 °C. Figure 2b shows that the weight loss rates of Stage 1 slags #1–4 were 0.54%, 0.38%, 0.53%, and 0.64%, respectively. Comparing #1–3, the weight loss rate decreases, but that of #3 is greater than that of #2, which is related to the Zn content. The weight loss rate of #4 is greater than that of #1–3. This trend is consistent with the loss trend of crystalline water. The above data show that reactions related to Zn and H_2_O occur before 900 °C, and the concentrations of both determine the volatilisation intensity in Stage 1.

The weight loss rates of slags #1–4 during Stage 2 were 0.38%, 1.08%, 3.5%, and 6.12%, accounting for 14.12%, 10.8%, 17.5%, and 15.3%, respectively, of the PbO content in the raw slag. The weight loss in Stage 2 corresponds to the PbO content. Comparing the volatilisation ratios of #1–3, #1 and #4, there is no direct relationship between the weight loss in Stage 2 and the ZnO content. However, whether there are indirect effects is worth exploring.

**Figure 3:**
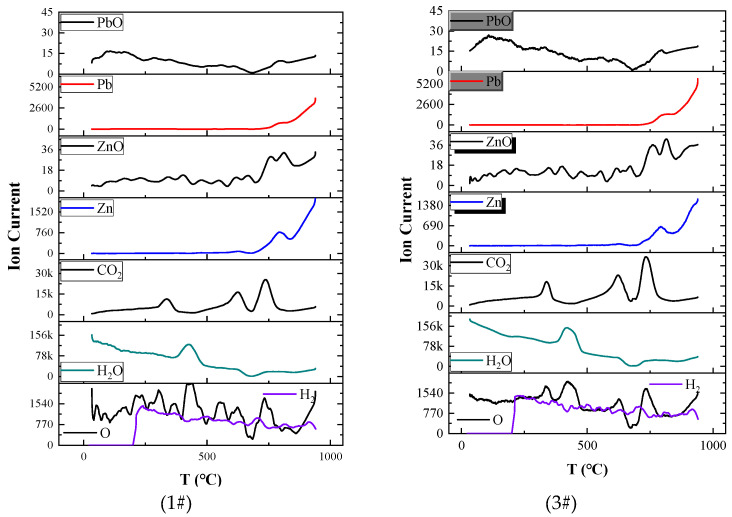
Plots of the ion currents of volatiles as a function of temperature for #1 and #3.

ISP-TOF can instantaneously detect substances that volatilise, thereby avoiding the effects of subsequent reactions on the original volatiles, and can approximately indicate the quantities of different reactants. The ISP-TOF detection results for substances released from samples #1 and #3 at elevated temperatures are depicted in Figure 3.

According to Figure 2, the strengths of the Pb and Zn curves were more than 100 times greater than those of the PbO and ZnO curves, respectively. It is deduced that the initial volatiles below 1000 °C consist of Pb(g) and Zn(g). In addition, PbO(g) examination is limited by the condensation of volatiles is also one of the reasons for the low strength of the PbO curve. For both substances, the curves for sample #1 closely align with those for sample #3 up to 800 °C, and display smaller and larger values between 800 and 1000 °C, respectively. Despite a sixfold increase in the PbO content of sample #3 compared to that of #1, the Zn(g) curve only shows a minor reduction. This suggests that while the ZnO content impacts the volatilisation volume to some extent, the PbO content does not significantly influence the generation or volatilisation of Zn(s).

Additional substances produced include H_2_O(g), CO_2_(g), O(g), and H_2_(g), with H_2_O(g) originating from the decomposition of CaCO_3_·xH_2_O between 370–480 °C. CO_2_(g) is generated within two temperature ranges: 310–370 °C and 560–790 °C. The reaction at 310–370 °C occurs when the low pressure of the TOF causes partial decomposition of CaCO_3_·xH_2_O in advance. O(g) has significant peaks at 310–480 °C and 700–790 °C, and the corresponding ion current of H_2_(g) gradually decreases, indicating that H_2_O(l) decomposes in small amounts. 

The above data indicate that the slight weight loss in Stage 1 is caused by Zn(g) and is related to H_2_O(s) in the slag. Zn(g) may transform into ZnO during volatilisation. This is basically the same as the result in Figure 2b.

Table 2 lists the saturated vapor pressures of Pb, Zn, PbO, and ZnO at 900–1500 °C [28]. According to Table 2, P_ZnO_ is very small, so ZnO(s) are not volatile. In addition, CaO, SiO_2_ and Fe_3_O_4_ are not volatile [29,30]. Hence, the volatiles in Stage 1 can be solely be Zn(s), indicating the necessity for a mechanism within the slag phase reaction that produces Zn(s). P_PbO_ increases 5 times after 900 °C, so the main volatile in Stage 2 is indeed PbO(g).

### 3.2. Tubular Furnace Experiment at High-Experiment

#### 3.2.1. The Maximum Volatilisation Ratio of Lead Slag

**Figure 4:**
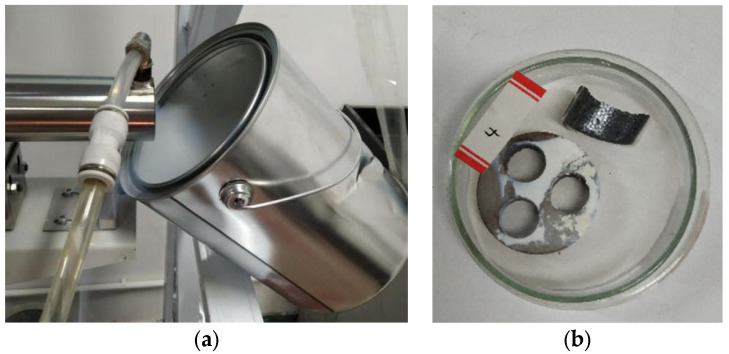
Volatile collection device and volatile matter morphology (**a**) Residual volatile gas after collection; (**b**) Collected substances and lead slag after reaction.

Direct tests of the volatile components can directly verify the results in Section 3.1. Hence, a scale-up experiment was performed to obtain a certain amount of volatiles using the device shown in Figure 1. Moreover, the holding time was extended to test the near maximum volatilisation ratio of the lead slag. Figure 4a shows the 5-condenser shell in Figure 1, which can collect approximately 95% of the volatiles, and the remaining 4–6% of the volatiles are deposited in the iron bucket. Figure 4b displays the metal disc with adsorbed white–yellow volatiles and the lead slag after the reaction.

Table 3 lists the weight loss rates of #1 through #4 when the samples were kept at 1350 °C for 8 h, where TWL refers to the total weight loss rate of the slag before and after volatilisation, and the calculation methods are shown in the formula in Table 3.

WAC represents the percentage of crystalline water and CO_2_ lost in the raw slag. This value is an estimate based on the weight loss rates of H_2_O and CO_2_ in Figure 2b. TWL-WAC is the volatilisation of PbO(s) and Zn(s) from the four types of slag. As shown in Table 3, the TWLs of #1 through #4 were 5.71%, 13.63%, 23.09%, and 32.53%, respectively. The TWL-WAC values for digesters #1 through #4 are 2.71%, 10.63%, 20.39%, and 29.33%, respectively. The TWL-WAC values of #1 through #3 exceed the sum of Stage 1 and Stage 2 in Figure 2b and exceed the percentage of PbO in the raw slag. This proves that ZnO(s) does contribute to the volatilisation rate. The volatilisation rate increases with increasing holding time until a certain limit is reached. The PbO in #1 to #3 is almost completely volatilised, and the PbO in #4 is not completely volatilised, remaining at approximately 25%, which explains why the volatile content impacts the limiting volatilisation rate.

From the results, it can be seen that there will be a great change of slag composition in the slag property measurement, such as viscosity, density, conductivity and so on. In these measurement process, the slag sample will be heated to a higher temperature over melting point and kept for a long time.

#### 3.2.2. Analysis of Volatiles

**Figure 5:**
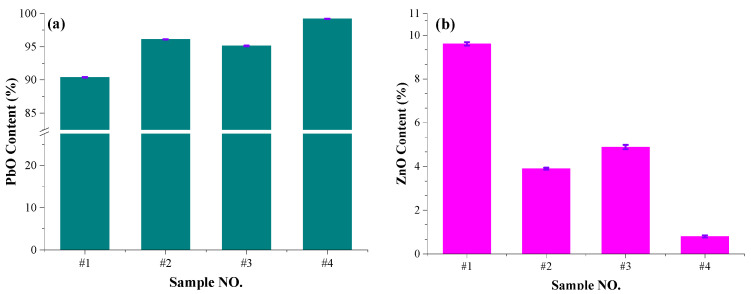
Composition changes in volatiles (**a**) PbO content of slags; (**b**) ZnO content of slags.

Figure 5 shows the results of volatile compound detection by ICPS.

Figure 5a,b reveal that as the PbO content increases and the ZnO content decreases, the Pb content in the volatile compounds reaches 90.39%, 96.1%, 95.12% and 99.2%, and the Zn content reaches 9.6%, 3.89%, 4.88% and 0.797%, respectively. The Pb contents of #1 to #4 are ordered as #1 < #3 < #2 < #4, and the Zn contents are ordered in reverse. The ratios of the Pb content to the Zn content vary between 9.4–124. The results is in accordance with Figure 2 and theoretical results of component vapor pressure in Table 2.

**Figure 6:**
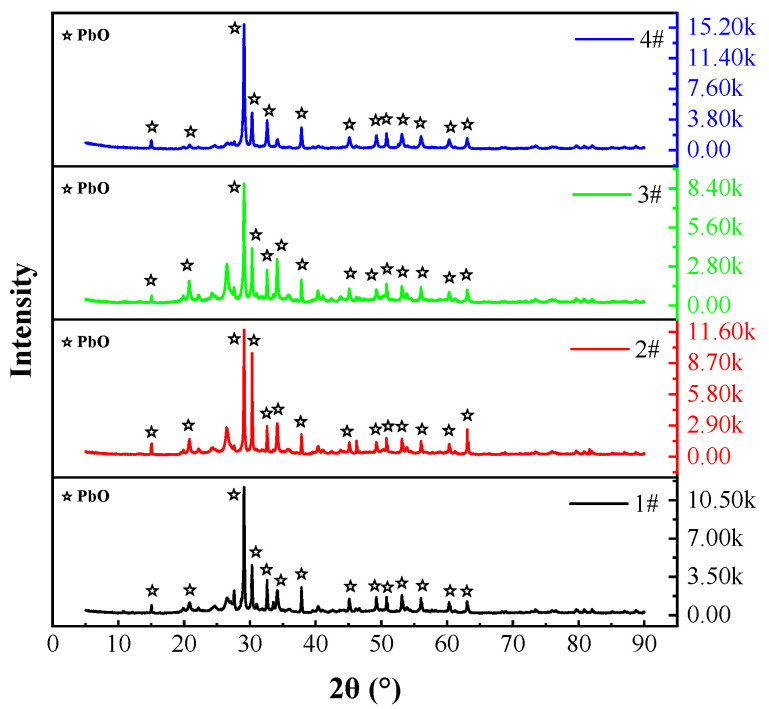
Physical phases of volatiles determined via XRD. #1, 2.69% PbO, 13% ZnO; #2, 10% PbO, 11% ZnO; #3, 20% PbO, 10% ZnO; #4, 40% PbO, 6% ZnO.

Figure 6 shows the results of XRD of the collected volatiles.

The collected volatiles of slags #1–4 are only PbO(s), and no substances containing Zn are detected. This can be attributed to the low content and poor crystallinity of Zn and ZnO. The above data indicate that the PbO content in slag is indeed a factor affecting volatilisation, PbO(s) are the main volatile component and Zn(s) are the secondary volatile component.

#### 3.2.3. Analysis of the Residual Slag Physical Phases

**Figure 7:**
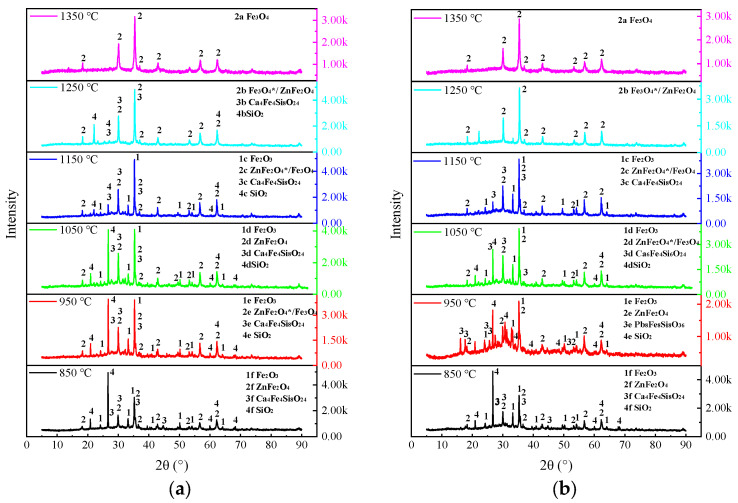

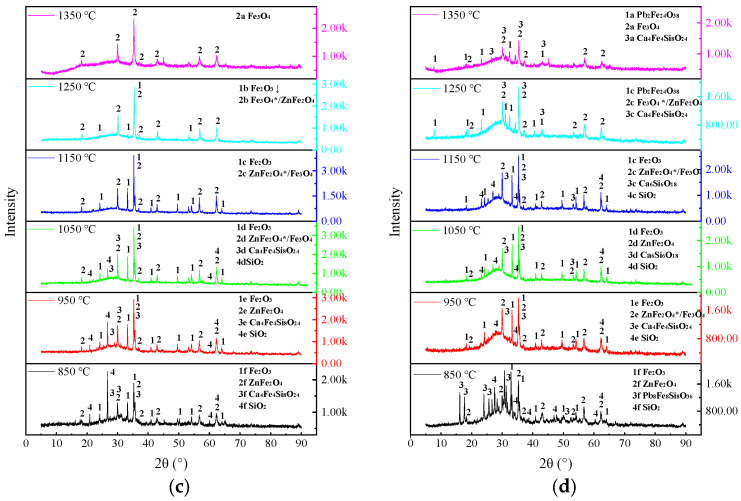
Physical phases of lead slag residue by XRD (**a**) #1, 2.69% PbO, 13% ZnO; (**b**) #2, 10% PbO, 11% ZnO; (**c**) #3, 20% PbO, 10% ZnO; (**d**) #4, 40% PbO, 6% ZnO.

Residual slag is the remaining of volatilisation tests. Figure 7 shows the XRD pattern of the residual slag from samples #1 to #4 at 850–1350 °C.

Upon comparing the four diagrams, it can be deduced that the variances among samples #1 to #4 in Figure 7 are characterised by the following observations: (a) The main peak intensities of the #1–4 XRD patterns decrease successively, indicating that an increase in the PbO content in slag will reduce the recrystallisation degree of slag. (b) The number of phases formed by recrystallisation of the four samples decreased when the temperature exceeded 1150 °C, and the physical phase at 1350 °C was dominated by Fe_3_O_4_(s). (c) The disappearance temperature of Ca_4_Fe_4_Si_8_O_24_(s) shows a trend of first decreasing and then increasing. (d) Pb_8_Fe_8_Si_8_O_36_(s) is present in samples #2 and #4, whereas Pb_2_Fe_24_O_38_(s) is found only in sample #4. Both compounds are predominantly formed from PbO and Fe_2_O_3_. 

The unique distributions of Ca_4_Fe_4_Si_8_O_24_(s) and Pb_8_Fe_8_Si_8_O_36_(s) across samples #1 to #4 suggest the presence of additional factors that influence the abnormal crystallisation of slag as the melting temperature decreases [30,31,32]. Based on the data pertaining to the remaining PbO in samples #1 to #4 as discussed in the third paragraph of Section 3.1, in addition to changes in heat capacity, volatilisation behaviour is identified as the primary cause. The extent of influence is directly proportional to the amount of volatiles. The interplay between PbO volatilisation and melting temperature contributes to the unusual findings in Figure 7, namely, at temperatures of 850 °C and 1150 °C, the order of crystallinity is #1 > #3 > #2 > #4; at other temperatures, the order is #1 > #2 > #3 > #4.

## 4. Conclusions

In this study, the volatilisation of PbO-ZnO-Fe_3_O_4_-SiO_2_-CaO slag at high temperature led to a number of conclusions.

1. The weight loss the slags is at the level of about 3.68% to 10.39%. when the temperature below 720°C, the weight loss caused by CO_2_ and crystalline water release exists. Above 900 °C, the weight loss can be considered the volatilisation of slag compononts and increases with the PbO content increase.
2. The volatile corresponding to the weight loss above 900 °C is mainly PbO (more than 90%) and less ZnO. The content of PbO in the collected volatile increases with the content of PbO in the slag. The content of ZnO in the collected volatile decreases with the content of PbO in the slag.
3. The higher the temperature is, the stronger the volatilisation is. With the increase in temperature and keeping time, most of the PbO can be evaporated and leaves little PbO in the residual slag. This will has great effect to physico-chemical property measurement of the slag with higher PbO content, especially to the property measurement that be kept at high temperature for a long time.
4. Volatile in-situ examination with pyrolytic time-of-flight (ISP-TOF) mass spectrometer is limited by the condensation of volatiles, i.e., PbO, Zn and so on, in the connection pipeline. The device should be modified for these use.

## Figures and Tables

**Table 1:** Lead slag ingredients.

No.	Component (%, ω)				
	PbO	ZnO	Fe_3_O_4_	CaO	SiO_2_
#1	2.69	13.00	44.97	11.24	28.10
#2	10.00	11.00	42.13	10.53	26.33
#3	20.00	10.00	37.33	9.33	23.33
#4	40.00	6.00	28.80	7.20	18.00

**Table 2:** Vapor pressures of Pb, Zn, PbO, and ZnO at different temperatures [10].

t/°C	T/K	P_Pb/_Pa	P_PbO/_Pa	P_Zn_/Pa___	P_ZnO_/Pa___
900	1173	4.224 × 10^1^	4.037 × 10^2^	0.932 × 10^5^	
1000	1273	1.858 × 10^2^	2.66 × 10^3^	2.334 × 10^5^	
1100	1373	6.552 × 10^2^	1.307 × 10^4^	5.078 × 10^5^	
1200	1473	1.937 × 10^3^	5.082 × 10^4^	9.878 × 10^5^	
1300	1573	4.968 × 10^3^	1.637 × 10^5^	1.756 × 10^6^	2.0 × 10^2^
1400	1673	1.134 × 10^4^	4.515 × 10^5^	2.901 × 10^6^	4.0 × 10^2^

**Table 3:** Weight loss of #1–4 when the samples were kept at 1350 °C for 8 h/%.

	#1	#2	#3	#4
Total weight loss rate (TWL)	5.71	13.63	23.09	32.53
Crystalline water and CO_2_ (WAC)	3.00	3.00	2.70	3.20
TWL-WAC	2.71	10.63	20.39	29.33

Formula: TWL = (Pre reaction mass − Post reaction mass)/Pre reaction mass × 100.

## Data Availability

Data are contained within the article.
